# Editorial: Cutting edge microscopy and imaging techniques in plant and algal research

**DOI:** 10.3389/fpls.2024.1496941

**Published:** 2024-10-07

**Authors:** Allison ML van de Meene, Elsa Arcalis

**Affiliations:** ^1^ School of Biosciences, Faculty of Science, University of Melbourne, Parkville, VIC, Australia; ^2^ Ian Holmes Imaging Centre, Bio21, The University of Melbourne, Parkville, VIC, Australia; ^3^ Institute of Plant Biotechnology and Cell Biology, Department of Applied Genetics and Cell Biology, University of Natural Resources and Life Sciences Vienna, Vienna, Austria

**Keywords:** microscopy - brightfield, microscopy - electron, multimodal imaging, multiscale imaging techniques, superresolution (sr), live cell imaging microscopy, microscopy-fluorescence

Microscopy and imaging techniques are powerful tools to study biological processes. These techniques are developing rapidly with technological innovations in hardware and improvements in image analysis algorithms and computing power. Imaging using microscopy techniques is based on light, fluorescence and electrons, where images are captured using a range of detectors that allow millimetre-to-micron-to-nanometre scale resolution. A combination of recent innovations has expanded spatial imaging to chemical and physical analytical domains, opening the door to a bigger picture. Using these techniques for studies on plant and algal research helps answer questions requiring whole plant, organ, tissue, cellular or macromolecular scales. This Research Topic on “Cutting-edge Microscopy and Imaging Techniques in Plant and Algal Research” showcases high-end imaging techniques and new developments in this research area.

## Live-cell, superresolution imaging

Live-cell imaging follows dynamic processes within living organisms and cells. When combined with superresolution imaging, complicated sample preparation techniques or high-end, specialised equipment is required. This is particularly true when attempting to combine live-cell, spatial superresolution and multicolour wavelengths at high temporal frequencies. Superresolution confocal live imaging microscopy (SCLIM) is one of the solutions to this dilemma, and recent advances have been reviewed by Ito et al. High speed SCLIM is based on spinning disk confocal technology, which overcomes the speed limitations of traditional laser scanning confocal techniques and allows the imaging of rapid and dynamic biological events. The addition of signal amplification for high-sensitivity detection improves the signal-to-noise ratio. Digital improvements using deconvolution algorithms brings the details into sharper focus again. The advantage of SCLIM over other techniques is the ability to observe dynamic cell processes at 30 frames per second (TV rate) using multiple wavelengths, and there is current, active, co-evolution of the technique using different approaches.

Live cell, superresolution microscopy, when combined with fluorescent protein technologies, is a powerful technique that can follow dynamic events at a resolution unachievable by traditional confocal laser scanning microscopes. Using current state-of-the-art Zeiss Airyscan technology, where the lateral resolution is 120 nm, McGinness et al. investigated the endoplasmic reticulum and Golgi connections in the *Nicotiana* and *Arabidopsis* experimental plant systems. From these experiments, connecting tubules between ER and Golgi bodies (ERGo tubules) that had not previously been observed were found. This research suggests that instead of vesicular anterograde transport, there are different forms of connections that traffic proteins between the ER and Golgi, a new insight that would remain hidden from the resolution of confocal laser scanning microscopy or the static images from ultrastructural electron microscopy.

Compared to using specialised instrumentation that enables superresolution imaging, Li et al. have used superresolution image reconstruction methods (SRIR) to improve the resolution of endocytotic vesicles from standard confocal micrographs. Two deep learning algorithms, superresolution generative adversarial networks (SRGAN) and superresolution residual networks (SRResNet), were trained on thousands of standard resolution confocal images and then applied to a test set of confocal data. The results improved the quality of the confocal images, identified the endocytic vesicle boundaries more clearly, improved the quantification of the vesicles and potentially unlocked deep mining of the information contained in these images. Overall, SRResNet appeared to perform slightly better for quantitative data and for deep mining while perceived image quality was better for the SRGAN data. This is a timely field of research given the rapid developments in artificial intelligence and the potential for how it might be applied to microscopy techniques.

## Multiscale and multimodal imaging

Imaging across multiple spatial scales (multiscale imaging) was used by Arcalis et al. to study the endomembrane system of maize endosperm tissue during seed development. The technically difficult live-cell imaging of the maize endosperm revealed the relevant role of vacuoles in the trafficking of seed storage proteins and highlighted the autophagic/autolytic nature of such vacuoles. The results were complemented with conventional transmission electron microscopy and serial block face scanning EM to build a three-dimensional model of the vacuoles, to gain a more complete picture of this compartment, its content and putative roles.

Like multiscale imaging, multimodal imaging incorporates different types of imaging into a single study. In the case of Permann et al., the project utilised multiscale imaging via optical and electron microscopy to investigate ultrastructure and further incorporated histological staining, confocal Raman spectroscopy and atomic force microscopy (AFM) imaging to analyse the conjugated sexual spores (zygospores) of the green alga *Spirogyra*. Each of these techniques adds unique information for a more comprehensive analysis of the samples. Histology and Raman confocal spectroscopy add to the chemical information known about the samples, and AFM provides insight into the physical properties and nanostructures of the cell wall revealing cell wall mechanics and adaptations to a non-aquatic environment.

Further refinement of multiscale, and an adaptation of multimodal imaging, was achieved by Lampugnani et al. In this study, the resolution of nano-scale imaging using electron microscopy was combined with the power of the molecular-genetic tag APEX2 and the accuracy of antibody labelling to confirm and clarify trafficking routes of cell wall polysaccharides and synthases in the model plant transformation system *Nicotiana benthamiana*. This study builds on the specificity of well characterised cell wall antibodies and the accurate localization of targeted protein tags. The results confirmed expected patterns of trafficking and revealed new insights in the cell biological mechanisms involved in the secretory process.

## Chemical imaging

Whole plant and organ research can be advanced by moving beyond the traditional microscopy techniques of optical, fluorescence and electron microscopy to obtain chemical information. Utilising scanning techniques similar to those used in fluorescent scanning confocal and scanning electron microscopy, spatially resolved, analytical techniques such as matrix-assisted laser desorption/ionization mass spectrometry (MALDI- MSI) can give insight into spatially resolved metabolite composition. Lin et al. have utilised the power of MALDI-MSI to spatially resolve the metabolite mescaline in the organs and flowers of ornamental cacti to characterize natural production of mescaline. Future applications are proposed to help in the detection of mescaline spiked plants couriered through the freight system.

## Conclusion

From these studies, it is evident that new and evolving techniques and technologies are opening up avenues of research that integrate multiple fields and produce cross-disciplinary research in the microscopy and imaging space. Alongside technological developments, improvements and adaptations in sample preparation, probes and image analysis will advance the field further. Future developments in bringing these threads together will progress our understanding of biological processes at different spatial and temporal scales.

